# N_2_O Hydrogenation on Silver Doped Gold Catalysts, a DFT Study

**DOI:** 10.3390/nano12030394

**Published:** 2022-01-25

**Authors:** José L. C. Fajín, Maria Natália D. S. Cordeiro

**Affiliations:** LAQV@REQUIMTE, Department of Chemistry and Biochemistry, Faculty of Sciences, University of Porto, P-4169-007 Porto, Portugal; ncordeir@fc.up.pt

**Keywords:** N_2_O elimination, greenhouse effect, bimetallic catalysts, heterogeneous catalysis, DFT calculations discipline

## Abstract

In this study, the full reaction mechanism for N_2_O hydrogenation on silver doped Au(210) surfaces was investigated in order to clarify the experimental observations. Density functional theory (DFT) calculations were used to state the most favorable reaction paths for individual steps involved in the N_2_O hydrogenation. From the DFT results, the activation energy barriers, rate constants and reaction energies for the individual steps were determined, which made it possible to elucidate the most favorable reaction mechanism for the global catalytic process. It was found that the N_2_O dissociation occurs in surface regions where silver atoms are present, while hydrogen dissociation occurs in pure gold regions of the catalyst or in regions with a low silver content. Likewise, N_2_O dissociation is the rate determining step of the global process, while water formation from O adatoms double hydrogenation and N_2_ and H_2_O desorptions are reaction steps limited by low activation energy barriers, and therefore, the latter are easily carried out. Moreover, water formation occurs in the edges between the regions where hydrogen and N_2_O are dissociated. Interestingly, a good dispersion of the silver atoms in the surface is necessary to avoid catalyst poison by O adatoms accumulation, which are strongly adsorbed on the surface.

## 1. Introduction

Nitrous oxide (N_2_O) is, together with CO_2_, CH_4_ and fluorine-containing halogenated substances (HFC_s_, PFC_s_, SF_6_ and NF_3_), one of the principal greenhouse gases emitted directly by human activities [1,2]. The warming potential of N_2_O is about 298 times larger than that of CO_2_ gas and 12 times larger than that of CH_4_, the emissions of this gas being around 7% (i.e., percentage based on MMT CO_2_ equivalents) of the greenhouse gases, and thus, the reduction of its emissions is compulsory [1]. In fact, the concentration of N_2_O in the atmosphere has increased about 23% since the preindustrial era.

Concerning the N_2_O sources, the activities related to agricultural soil management are the main source of anthropogenic N_2_O, currently being about 75% of the unnatural N_2_O emissions in U.S.A. [1]. Other human activities, such as wastewater treatment, fertilizer management, stationary and mobile combustion, and nitric acid production, also contribute considerably to these gas emissions [1].

Furthermore, the most convenient way for N_x_O_y_ oxides elimination is through its catalytic reduction to atmospheric N_2_ on solid catalysts. Solid catalysts in the form of nanoparticles are extensively used in catalysis, such as in reactions driving the elimination of atmospheric or water pollutants or hydrogen production [3,4,5]. The techniques used in the catalysis of this reaction are usually grouped into two generic reaction schemes, that is, selective catalytic reaction (SCR) [6] and the NO_x_ storage-reduction (NSR). SCR, which is usually used in stationary N_x_O_y_ sources, consists of the direct reduction of N_x_O_y_ oxides on the catalyst usually assisted by a reducing agent, such as NH_3_, urea, or hydrocarbons [7,8,9]. The NSR process, in contrast, is carried out in two cycles: (i) in the long oxygen-rich cycle, the N_x_O_y_ species are captured and stored, and (ii) in the short fuel cycle, the N_x_O_y_ species are reduced to N_2_ on the catalyst [10,11]. Therefore, the NSR technique is appropriate for the reduction of N_x_O_y_ oxides produced by combustion engines. One should note here that additional N_2_O can be produced during the reduction of other N_x_O_y_ oxides; consequently, it is necessary to avoid the side reactions leading to such species [12,13,14,15].

A wide variety of solid catalysts was proved experimentally with success in the reduction of N_x_O_y_ oxides to N_2_, such as the commercial V_2_O_5_−WO_3_(MoO_3_)/TiO_2_ catalyst that is used in thermal power plants due to its high efficiency in the removal of nitrogen oxides in the temperature range of 300 °C to 400 °C [16]. However, this catalyst suffers from deactivation by the poisoning of several species which are present in the effluent gases, and also, it cannot be used neither in plants where the effluent gases temperature is lower than 300 °C nor in diesel engines [9]. Other metal oxide based catalysts were also probed with success in the catalytic reduction of nitrogen oxides to N_2_, such as those based on VO_x_, MnO_x_, CeO_2_, Fe_2_O_3_ or CuO, as well as acid compound catalysts, ion exchange zeolite catalysts or a monolith catalyst [7,9]. Additionally, the catalyst activity for the NO_x_ species reduction at low temperatures can be increased, using a bimetallic Ag–Au alloy supported on Al_2_O_3_, where silver species are partially positively charged and well dispersed [17,18]. The formation of additional N_2_O gas during the NO_x_ species reduction in the bimetallic catalyst is related to the presence of large silver clusters, while for the Ag–Au alloy, the formation of this species was not detected [17,18].

Regarding the merely N_2_O reduction on solid catalysts, transition metals, such as Co, Cu, Fe, Zr, and Ni dispersed on CeO_2_ or forming a mixed oxide with this species, were probed with success in the catalysis of that reaction, varying the temperature range for the reduction between 300 °C and 660 °C [19,20,21,22]. More recently, Jacobs et al. [23] also employed Ag–Au alloys to dissociate N_2_O into N_2_ and atomic oxygen, the latter species being then further hydrogenated with H_2_ to yield water. The temperature range considered in their experiments is from 300 K to 320 K, which is considerably lower than that usually used in NO_x_ reduction [23]. The authors also found that the N_2_O hydrogenation on the Ag–Au alloy is highly structure sensitive and that the active sites for the N_2_O and H_2_ dissociation are those where the (210) facets are present. Furthermore, the preferential dissociation sites were shown to be independent of the reactants’ pressure ratios, thus suggesting a Langmuir–Hinshelwood mechanism for the N_2_O hydrogenation reaction on Ag–Au alloys [23]. In addition, the presence of silver atoms surrounded by gold atoms prevented the excessive accumulation of oxygen atoms on the surface that can poison the catalytic surface [23]. The reactivity of Ag–Au alloys for the N_2_O hydrogenation can be influenced by the silver segregation toward the surface, but, in the case of the surface exposure to N_2_O, this is lower than if the surface is exposed to O_2_ gas [24].

In addition, bimetallic Ag–Au based catalysts also present a high catalytic activity for oxidative reactions, such as methanol coupling [25,26] or CO oxidation [27], where the oxygen activation is the key reaction step [28,29]. Moreover, atmospheric contaminants such as CO can be also eliminated through its hydrogenation toward valuable products, such as methane or higher hydrocarbons or alcohols [30,31].

In this work, we further the understanding of the N_2_O reduction by hydrogen on Ag–Au alloys through the investigation of the reaction mechanism at the atomistic level. To do that, the N_2_O and H_2_ dissociations, the N_2_ formation and the O hydrogenation toward water are theoretically studied on a model representing a portion of the bimetallic catalytic alloy with the (210) termination, just as it was experimentally confirmed. One should remark here that theoretical methods can provide a detailed atomic-level description of both the catalysts active sites, reaction mechanisms and synergic effects, which are crucial factors to reveal the catalytic properties of experimental catalysts [32].

## 2. Materials and Methods

### 2.1. Reaction Mechanism

The N_2_O dissociation and further O hydrogenation on Ag@Au bimetallic alloy can evolve through a series of steps, which are listed below.

In accordance with the experimental evidence, the N_2_O hydrogenation follows a Langmuir–Hinshelwood mechanism [23]; thus, the reactants adsorb on the catalytic surface before they suffer their respective surface reactions. That is, the first steps of the process are N_2_O and H_2_ adsorption:

(1) $$N_{2}O+\;⊗\;\to N_{2}O^{*}$$

(2) $$H_{2}+\;⊗\;\to H_{2}{}^{*}$$

where ⊗ denotes a free adsorption site, while * denotes species adsorbed on the surface.

After the reactants’ adsorption, their dissociations on the surface occur, leading to N_2_ + O and H + H also being adsorbed on the surface:

(3) $$N_{2}O^{*}\;\to N_{2}{}^{*}+O^{*}$$

(4) $$H_{2}{}^{*}\to H^{*}+H^{*}$$

According to our calculations, N_2_ formed from N_2_O dissociation easily desorbs from both surfaces considered in this work.

(5) $$N_{2}{}^{*\;}\to N_{2}+\;⊗$$

On the other hand, the O* species from the nitrous oxide dissociation reacts with H* from hydrogen dissociation to form a hydroxyl species on the surface which suffers new hydrogenation, yielding water.

(6) $$O^{*}+H^{*}\;\to {\mathrm{OH}}^{*}$$

(7) $${\mathrm{OH}}^{*}+H^{*}\;\to H_{2}O^{*}$$

After the water formation, the latter desorbs from the surface.

(8) $$H_{2}O^{*\;}\to H_{2}O+\;*$$

### 2.2. Catalytic Surfaces Modeling

To investigate the N_2_O hydrogenation on bimetallic Ag@Au surfaces with the (210) termination, we began by firstly establishing which of the later types of terminated surfaces are the most stable. For such purpose, bimetallic Ag@Au(210) surfaces with different silver concentrations were optimized. Surfaces with (210) termination are stepped surfaces with a straight step, where low coordinated atoms are present as in other surface defects, and those are usually related to the presence of low coordinated atoms in catalytic surfaces with the promotion of dissociative reaction steps. One should notice here that, even though the former experiments carried out for examining the N_2_O hydrogenation catalysis resorted to a catalyst based on an Ag@Au alloy with a 8.8% of silver content, the data gathered do not give us the exact concentration of silver atoms in the top layers of the surface because Ag atoms can segregate to the surface or in the opposite direction [23]. In spite of this, the Ag segregation does not look like a crucial aspect to take into account when the surface is exposed to N_2_O [24]. Importantly, silver atoms are well dispersed in the catalytic surfaces of the experimental catalyst, avoiding the disposition of an excessive accumulation of surface oxygen atoms and catalyst poisoning by this species [24].

Bimetallic surfaces used in this work are modeled through the periodic repetition of a slab of adequate symmetry; this slab represents a portion of the catalyst and, through its periodic repetition, it is avoided the boundary problem which appears if a finite system is considered. Vesta program [33] was used to obtain the initial positions of the atoms in the slab with the (210) termination whose dimensions correspond to a 2 × 2 cell, with respect to the minimal unit cell for those Miller indices, in the *x* and *y* directions, while in the *z* direction, four atomic layers are considered. Further, a vacuum region with a thickness of 10 Å is introduced in the z direction to generate the surfaces by separation of the slab replicas in that direction. The thickness of the vacuum region is considered to be enough to avoid the interaction of adsorbates on the surface with the bottom part of the next replica.

**Figure 1:**
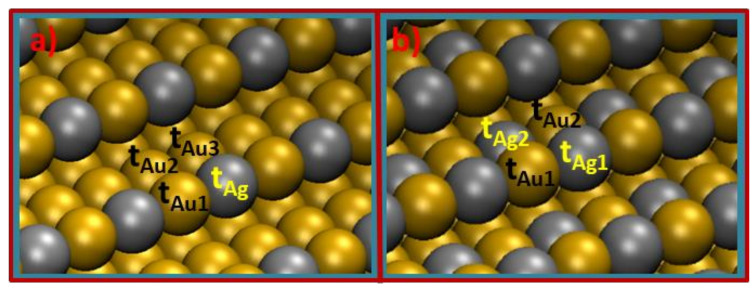
Representation of the Au(210) surface doped with (**a**) one silver atom per unit cell in the step (Ag@Au(210) surface) and (**b**) one silver atom per unit cell in the step and another in the terrace (Ag_2_@Au(210) surface). Top adsorption positions are indicated by *t_Ag_* for Ag and *t_Au_* for Au atoms, while the adsorption in bridge positions is indicated by *b* followed of the name used for the atoms forming the bridge in subscripts. Adsorption position in hollow formed by the four atoms shown in both panels of this figure is indicated by *h_step_*. Silver atoms are colored in grey, while gold atoms are colored in ochre.

Moreover, bimetallic alloyed surfaces are generated by replacing matrix gold atoms in the slab by silver atoms. We performed a geometrical optimization of the different surfaces that can be obtained by the replacement of one or two gold atoms in symmetrically inequivalent positions of the top layer of the slab by silver atoms, and the most stable surfaces for one and two atoms’ substitutions were further used in the calculations (see Figure 1 for optimized configurations used in the calculations and adsorption positions on them.). Finally, the bulk metal was simulated by keeping frozen the positions of atoms in two bottom metallic layers, while the positions of atoms in two topmost metallic layers and adsorbates were entirely relaxed. From now on, the most stable surface generated by the replacement of one gold atom by a silver atom in the top layer of the slab is denoted as Ag@Au(210), while that where two gold atoms are substituted by silver atoms is denoted as Ag_2_@Au(210). The former corresponds to a replacement in the surface step, while the latter corresponds to substitutions in the step and in the terrace.

### 2.3. Computational Details

Following the mechanism for the N_2_O hydrogenation comprising the reaction steps given in Equations (1)–(8), the most stable adsorption configurations and energies for the reactants and products adsorption are described in this section, as well as the calculation of the activation energies and rate constant for each such reaction step.

The most stable adsorption configurations and their respective electronic energies are obtained through energy minimization with respect to the geometry of adsorbates (reactants or products of the reaction steps) on all the adsorption positions of the surfaces mentioned in the previous section (i.e., on Ag@Au(210) and Ag_2_@Au(210) surfaces). To do so, spin polarized periodic DFT calculations were carried out with the VASP computer code [34,35,36], considering the PBE generalized gradient approach (GGA) exchange correlation potential [37] to take into account the electron density of the systems. The PBE density functional is selected due to its good performance for calculations involving reactions on surfaces [38]. Van der Waals corrections were introduced in the calculations following the D3 method due to Grimme et al. [39]. Further, the valence electronic states of reactants and slab atoms were described using a set of plane waves, while the electronic states for core electrons were described using the projected augmented-wave (PAW) method [40,41], considering the effect of the core electrons in the valence electron density through the latter method. Moreover, the expansion of the kinetic energy of the plane waves was limited by a cutoff of 415 eV, while the numerical integration in the reciprocal space was done considering a set of special *k*-points established by the application of a 7 × 7 × 1 Monkhorst-Pack grid [42]. The coordinates of the adsorbates’ atoms and the two topmost metallic layers of the surface were relaxed during the calculations, using a conjugate gradient algorithm. However, the coordinates of the atoms in the two bottom metallic layers were kept frozen to simulate the metallic bulk. The convergence criteria used in the geometry optimizations were 10^−6^ eV for the total energy change and 10^−3^ eV/Å for the forces acting on the atoms, which are very strict criteria, preventing the algorithm from stopping in the local minima. Moreover, the convergence of the calculations with respect to all these parameters was fully checked for selected configurations.

For determining the activation energy of each reaction step, the corresponding transition states (TS) joining reactants with products were located using the Dimer method [43], which is also implemented in the VASP code. This computational approach is very versatile, as previous knowledge of the approximate reaction path is not necessary to start the calculations. The convergence criteria used in the TS search were the same as those in the geometry optimizations. Further, the TS structures obtained were further confirmed through the analysis of their vibrational frequencies, and by corroborating that the movement associated with the single imaginary frequencies found drives the systems from reactants to products.

Adsorption energies of the optimized geometries were obtained as follows:

(9) $$E_{ads\left(i\right)}^{ZVPE}=E_{slab+adsorbate\left(i\right)}^{ZVPE}-\left(E_{slab}+E_{adsorbate\left(i\right)}^{ZPVE}\right)$$

where *i* represents the adsorbate(s) being considered, $E_{ads\left(i\right)}^{ZVPE}$ is the adsorption energy of the species *i* including the zero-point vibrational energy corrections (*ZPVE*), $E_{slab+adsorbate\left(i\right)}^{ZPVE}$ is the electronic energy plus the *ZPVE* corrections of the slab-adsorbate(*i*) system, $E_{slab}$ is the electronic energy of the slab, and $E_{adsorbate\left(i\right)}^{ZPVE}$ is the gas phase electronic energy of the *absorbate(i)*, including the *ZPVE* corrections.

Zero-point vibrational energy corrections of each state were obtained following the harmonic oscillator approach, considering the vibrational frequencies of the correspondent system, that is,

(10) $$E_{system}^{ZPVE}=\frac{1}{2}\underset{i}{\sum }h\nu_{i}$$

where *ν_i_* stands for the frequencies of vibrational modes of the system (slab + adsorbate(s) or adsorbate(s)) on the surface or on gas phase and *h* is the Planck constant.

According to Equations (9) and (10), negative values of *E_ads_* mean favorable adsorptions.

Further, reaction energies $E_{react}^{ZPVE}$ for all the reaction steps given in Equations (1)–(8) were obtained, subtracting to the final state energy (FS: *slab + products*) that of the initial state (IS: *slab + reactants*) and also taking into account the *ZPVE* corrections. That is,

(11) $$E_{react}^{ZPVE}=E_{FS\left(slab+products\right)}^{ZPVE}-E_{IS\left(slab+reactants\right)}^{ZPVE}$$

Activation energy barriers ($E_{act}^{ZPVE}$) for the reaction steps given in Equations (1)–(8) were determined as the energy difference between that of the TS and that of the IS (*ZPVE* corrections are also taken into account in their determination):

(12) $$E_{act}^{ZPVE}=E_{TS}^{ZPVE}-E_{IS}^{ZPVE}$$

Finally, an estimation of the rate constants (*k*) for the reaction steps given in Equations (1) to (8) was done by assuming valid the classical transition state theory [44], i.e., as follows:

(13) $$k=\left(\frac{k_{B}T}{h}\right)\left(\frac{Q^{\neq }}{Q}\right)e^{-E_{act}^{ZPVE}∕k_{B}T}$$

where $Q^{\neq }$ and Q are the vibrational partition functions of the TS and the IS states, respectively, which were calculated from the vibrational frequencies of the respective states. $E_{act}^{ZPVE}$ is the activation energy barrier calculated following Equation (12), *k*_B_ is the Boltzmann constant, and *h* the Planck constant. *T* is the absolute temperature that was set to values of 200, 225, 250, 275, 300, 325, 350 and 400 K. The temperature range was chosen according to the values used in the experiments concerning N_2_O hydrogenation on Ag@Au-based catalysts [23].

### 2.4. Experimental Observations and Knowledge Lacks

Bimetallic Ag@Au catalyst presents a high activity for N_2_O hydrogenation at low temperature (300 to 320 K) [23], this feature being very important due to the lack of adequate catalysts for the catalysis of that reaction at low temperatures. Experimental observations revealed the formation of a reactive interface around the periphery of the (210) facets, where water is supposed to be formed by the reaction of O* adatoms with H* adatoms [23]. The formation of the H* and O* interface is independent of the partial pressures of the reactants, thus suggesting a Langmuir–Hinshelwood mechanism for the N_2_O hydrogenation reaction on the Ag@Au catalyst. Moreover, the formation of this interface with O* and H* adatoms also suggests that H_2_ and N_2_O dissociations take place in specific sites of the catalytic surface, when assuming that the N_2_O dissociation occurs on the (210) facets [23]. The surface reconstruction during the reaction or after the change of the reaction conditions is not detected. Further, silver segregation toward the surface after the exposition of that to N_2_O gas is predicted to be a not very important aspect for Ag@Au surfaces [24].

Despite this experimental knowledge, there are several aspects related with the N_2_O catalytic hydrogenation on Ag@Au surfaces that have to be clarified. First of all, it is not clear which are the dissociation sites for H_2_ and N_2_O species, nor if the spillover of some species toward the (210) facets edge is necessary previous to water formation. What is more, the exact composition of the catalytic surface is also not known nor is the role of the silver atoms in the catalysis of each reaction step. Following this, the silver dispersion in the catalytic surface should be also an important factor in the catalysis because it is well known that O* adatoms adsorb strongly in pure silver surfaces. Finally, the exact mechanism of the N_2_O hydrogenation on Ag@Au surfaces is also not known, nor is the rate determining step for that reaction.

## 3. Results

In this section, the most relevant results for the study of N_2_O hydrogenation on Ag_x_@Au(210) surfaces are presented and discussed. Firstly, the most stable adsorption configurations and energies for all the species involved in that reaction are given and discussed. Thereafter, the most favorable reaction paths for each step are established, followed by the global reaction mechanism inferred from the energetic quantities of the individual steps.

### 3.1. Adsorption of Reactants and Products

To establish the most favorable mechanism for N_2_O hydrogenation on Ag_x_@Au(210) surfaces, it is necessary to locate for each reaction step the most favorable adsorption geometries of the whole involved reactant and product species.

**Figure 2:**
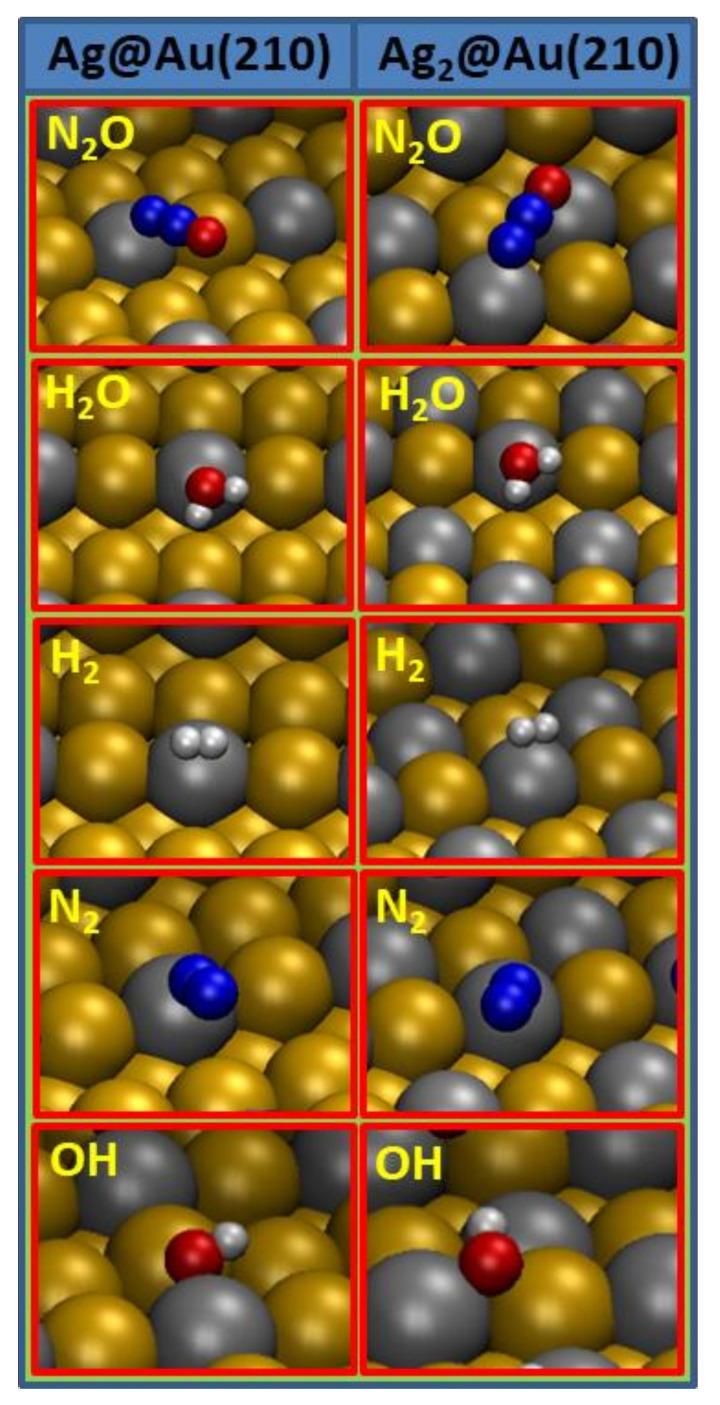
Most stable configurations for the adsorption of individual species on Ag@Au(210) and Ag_2_@Au(210) surfaces, which are involved in the N_2_O hydrogenation. Red color is used for oxygen, white for hydrogen, blue for nitrogen, grey for silver and ochre for gold atoms.

**Figure 3:**
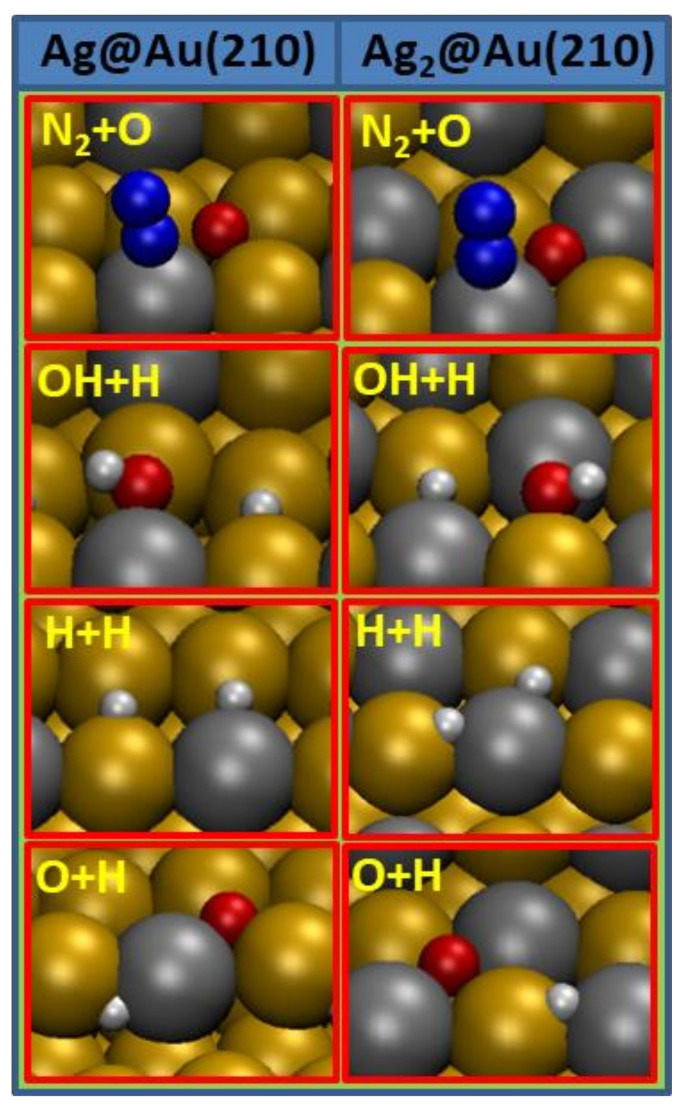
Most stable configurations for the coadsorption of pairs of species on Ag@Au(210) and Ag_2_@Au(210) surfaces which are involved in the N_2_O hydrogenation. Red color is used for oxygen, white for hydrogen, blue for nitrogen, grey for silver and ochre for gold atoms.

Reactants and products of reaction steps given in Equations (1)–(8) can be grouped as individual species adsorbed on the surfaces and pairs of species coadsorbed on the surfaces, namely, N_2_O, H_2_O, N_2_, H_2_, OH and N_2_ + O, OH + H, H + H, and O + H. All the results for the individual species adsorption (adsorption positions and energies; internal and absorbate to surface distances; vibrational modes) on the Ag@Au(210) and Ag_2_@Au(210) surfaces are given in Table 1, while those for the pairs’ adsorption are given in Table 2. The representation of the most favorable adsorption configurations for these species or pairs on the Ag@Au(210) and Ag_2_@Au(210) surfaces can be seen in Figure 2 and Figure 3, respectively.

Analyzing the results given in Table 1 for the adsorption of individual species (N_2_O, H_2_O, N_2_, H_2_ and OH) on the Ag@Au(210) and Ag_2_@Au(210) surfaces, it can be seen that the energetic quantities for the adsorptions on the Ag@Au(210) surface are close to those on the Ag_2_@Au(210) surface. This result shows that the crucial aspect in the adsorption of individual species is the type of atom/s in the surface interacting with the adsorbate, showing the local character of the adsorption of these species. Note that most of the species are preferably adsorbed on the Ag_x_@Au(210) surfaces interacting with the silver atoms in the step, and therefore, the inclusion of more silver atoms in the surface terrace does not significantly alter the stretch of the bond(s) between the absorbate and the surface. Apart from the adsorption energy values of both surfaces, the distances between the absorbate and the surface are also similar on Ag@Au(210) and Ag_2_@Au(210) surfaces for the same species adsorption, as well as the frequencies for the vibrational modes.

We move on now with the analysis of the adsorption of each species. N_2_O adsorbs by the terminal *N* atom in *t_Ag_* site of the Ag@Au(210) surface, while it is adsorbed through terminal *N* and *O* atoms in *b_Ag1–Ag2_* site of the Ag_2_@Au(210) surface. The adsorption energy of both surfaces is about ~−0.3 eV, a moderate-low value, which predicts an important role of the desorption during the N_2_O hydrogenation. Further, water adsorption occurs on Ag_x_@Au(210) surfaces preferably on a silver atom of the step through the *O* atom, the molecular plane being parallel to the surface terrace. Water adsorption energy is about ~−0.5 eV for both surfaces; consequently, the adsorption of this species is not notably influenced by the silver content of the surface. H_2_ adsorption occurs on Ag_x_@Au(210) surfaces, preferably also on a silver atom of the step in both surfaces, and, being only physisorbed, again, the silver content in the surface does not alter considerably the adsorption energy for molecular hydrogen. N_2_ adsorption follows a similar trend; it is preferably adsorbed on silver atoms of the Ag_x_@Au(210) surfaces steps, and the adsorption energy is about ~−0.15 eV for both surfaces. Hydroxyl adsorption is more favorable in bridge positions, where silver atoms are present; in the case of the Ag_2_@Au(210) surface, this adsorption occurs preferably in bridge positions of the step, whereas, in the case of the Ag@Au(210) surface, it occurs in bridge positions of the terrace. The adsorption energy for hydroxyl is about ~−2.3 for both surfaces.

**Table 1:** Adsorption energies ($E_{ads}^{ZPVE}$, eV), vibrational wavenumbers and bond lengths (d, Å) for the species involved in the N_2_O hydrogenation on Ag@Au(210) and Ag_2_@Au(210) surfaces.

**Ag@Au(210) Surface**					
**Species**	**Adsorption Site ^a^**	$E_{ads}^{ZPVE}$	**Vibrational Wavenumbers ^b^**	d_suf-mol_^c^	Bond Length ^d^
**N_2_O**	*t_Ag_*	−0.33	2340; 1331; 542; 536	2.56 (N–Ag)	1.15 (N–N) 1.19 (N–O)
**H_2_O**	*t_Ag_*	−0.50	3730; 3442; 1556	2.46 (O–Ag)	0.98 (O–H_b_) 0.99 (O–H_a_)
**H_2_**	*t_Ag_*	−0.06	526	2.43 (H_a_–Ag)/2.46 (H_b_–Ag)	0.76 (H_a_–H_b_)
**N_2_**	*t_Ag_*	−0.15	2416	2.72 (N–Ag)	1.12 (N–N)
**OH**	*b_Ag-Au3_*	−2.29	3698; 794	2.22 (O–Ag)/2.15 (O–Au3)	0.98 (O–H)
**Ag_2_@Au(210) Surface**					
**Species**	**Adsorption Site ^a^**	$E_{ads}^{ZPVE}$	**Vibrational Wavenumbers ^b^**	d_suf-mol_^c^	Bond Length ^d^
**N_2_O**	*b_Ag1-Ag2_*	−0.29	2337; 1327; 536; 531	2.78 (N–Ag1)/3.34 (O–Ag2)	1.15 (N–N) 1.20 (N–O)
**H_2_O**	*t_Ag1_*	−0.47	3737; 3409; 1560	2.47 (O–Ag1)	0.98 (O–H_b_) 0.99 (O–H_a_)
**H_2_**	*t_Ag1_*	−0.03	540	2.63 (H_a_–Ag1)/2.64 (H_b_–Ag1)	0.76 (H_a_–H_b_)
**N_2_**	*t_Ag1_*	−0.17	2422	3.08 (N–Ag1)	1.12 (N–N)
**OH**	*b_Ag1-Au1_*	−2.27	3710; 743; 518	2.23 (O–Ag1)/2.15 (O–Au1)	0.98 (O–H)

^a^ The notation used for adsorption sites is shown in Figure 1. ^b^ Only the vibrational modes above 500 cm^−1^ are given. ^c^ Distances from the adsorbed species to the surface; between parentheses, it is indicated the atom/s through the adsorbate and the surface interact. ^d^ Lengths of the internal species bonds, being indicated between parentheses the correspondent bond to each length.

On the other hand, the coadsorption of several pairs of species was also studied on Ag_x_@Au(210) surfaces because these are the IS or FS of several reaction steps involved in N_2_O hydrogenation. Comparing the entries given in Table 2 for the N_2_ + O, OH + H, H + H, and O + H coadsorption on the Ag_x_@Au(210) surfaces, the same conclusion as that obtained for the individual species adsorption arises, i.e., the increase in silver content in the surface does not significantly alter the adsorption energies of these pairs. This is due to the preferable interaction of the absorbates with the silver atoms of the step where the silver content is similar in both surfaces. Further, preferably adsorption sites, distances and vibrational frequencies are similar for each pair in both surfaces. What is more, the coadsorption of all the pairs is very favorable with respect to the fragments in the gas phase for both surfaces, presenting large adsorption energies which will prevent the desorption of these species during N_2_O hydrogenation.

Finally, there are not comparable theoretical or experimental results for the adsorption on Ag_x_@Au(210) surfaces, nor on the pure Au(210) surface of the species studied in this work. Therefore, a direct comparison with previous results cannot be done; only an approximate comparison with the results for the adsorption of the same species in similar metallic surfaces is possible. For instance, the N_2_O adsorption was studied on the pure Au(321) surface, using a similar methodology [45]; the adsorption of this species does not occur on the Au(321) surface, while the H_2_ is only physisorbed. On the surfaces considered in this work, the N_2_O is adsorbed in the silver atoms with adsorption energy values of about ~−0.3 eV, while H_2_ is physisorbed as in the pure Au(321) surface. Moreover, the N_2_ species is slightly more strongly adsorbed on the surfaces studied here than on the pure Au(321) surface [45]. Summing up, the doping of gold surface with silver atoms seems to have a crucial role in N_2_O adsorption, but it is not as important in N_2_ or H_2_ adsorption.

In contrast, water and hydroxyl adsorption were extensively studied both in pure metallic [46,47] or bimetallic surfaces [48]; for instance, the water adsorption energy has values of −0.18 eV on Au(111) surface, −0.31 eV on Ag(111), −0.34 eV on Au(321), −0.67 eV on Ni@Au(110), etc. The values obtained in this work for water adsorption, −0.50 eV on Ag@Au(210) and −0.47 eV on Ag_2_@Au(210) surface, are considerably higher (in absolute value) than those obtained in pure silver or gold surfaces, showing the importance of the surface doping in the water adsorption. Hydroxyl is strongly adsorbed in all the metallic surfaces previously studied [46,47,48], as it was also obtained for the Ag_x_@Au(210) surfaces.

### 3.2. Activation Energy Barriers and Rate Constants for the Steps Involved in the N_2_O Hydrogenation

The N_2_O hydrogenation on Ag_x_@Au(210) surfaces encompasses the reaction steps shown in Section 2.1 (see Equations (1)–(8)). One then needs to find the most favorable path for those steps to clarify its mechanism. To that end, the IS and FS for each reaction step were determined by examining the adsorption of the corresponding reactants or products (see Section 3.1), and the relevant TS located following the computational strategy outlined in Section 2.3.

**Table 2:** Adsorption energies ($E_{ads}^{ZPVE}$, eV), vibrational modes (cm^−1^), and structural parameters (d, Å), for the co-adsorption of pairs of species involved in the N_2_O hydrogenation on Ag@Au(210) and Ag_2_@Au(210) surfaces.

**Ag_2_@Au(210) Surface**					
**Species**	**Adsorption Site ^a^**	$E_{ads}^{ZPVE}$	**Vibrational Wavenumbers ^b^**	d_suf-mol_^c^	Bond Length ^d^
**N_2_ + O**	*t_Ag/_h_step_*	−3.50	2426	2.53 (N–Ag)/2.67 (O–Ag) /2.28 (O–Au1) /2.13 (O–Au2) /2.19 (O–Au3)	1.12 (N–N)
**OH + H**	*b_Ag-Au3/_b_Au1-Au2_*	−4.65	3691; 1234; 1117; 824	2.21 (O–Ag)/1.76 (H–Au1) 2.15 (O–Au3)/1.77 (H–Au2)	0.98 (O–H_a_)
**H + H**	*b_Ag-Au3/_b_Au1-Au2_*	−4.19	1566; 1242; 1094; 767	1.93 (H_a_–Ag)/1.75 (H_b_–Au1) 1.68 (H_a_–Au3)/1.77 (H_b_–Au2)	–
**O + H**	*h_step_/b_Ag-Au1_*	−5.45	1661; 1065	2.12 (O–Ag)/1.82 (H–Ag) 2.04 (O–Au1)/1.77 (H–Au1) 2.35 (O–Au2)/ 2.15 (O–Au3)/	–
**Ag_2_@Au(210) Surface**					
**Species**	**Adsorption Site ^a^**	$E_{ads}^{ZPVE}$	**Vibrational Wavenumbers ^b^**	d_suf-mol_^c^	Bond Length ^d^
**N_2_ + O**	*t_Ag/_h_step_*	−3.52	2425	2.78 (N–Ag1)/2.32 (O–Ag1) /2.19 (O–Au1) /2.26 (O–Au2) /2.31 (O–Ag2)	1.12 (N–N)
**OH + H**	*b_Ag1-Au2/_b_Au1-Ag2_*	−4.65	3681; 1588; 843; 688	2.21 (O–Ag1)/1.68 (H–Au1) 2.13 (O–Au2)/1.93 (H–Ag2)	0.98 (O–H_a_)
**H + H**	*b_Ag1-Au1/_b_Ag1-Au2_*	−4.25	1675; 1569; 665; 600	2.01 (H_a_–Ag1)/1.97 (H_b_–Ag1) 1.66 (H_a_–Au1)/1.68 (H_b_–Au2)	—
**O + H**	*h_step_/b_Ag1-Au1_*	−5.48	1573; 829	2.27 (O–Ag1)/1.88 (H–Ag1) 2.33 (O–Ag2)/1.68 (H–Au1) 2.30 (O–Au1)/ 2.20 (O–Au2)/	—

^a^ The notation used for adsorption sites is shown in Figure 1. ^b^ Only the vibrational modes above 500 cm^−1^ are given. ^c^ Distances from the adsorbed species to the surface; between parentheses, it is indicated the atom/s through the adsorbate and the surface interact. ^d^ Lengths of the internal species bonds, being indicated between parentheses the correspondent bond to each length.

**Figure 4:**
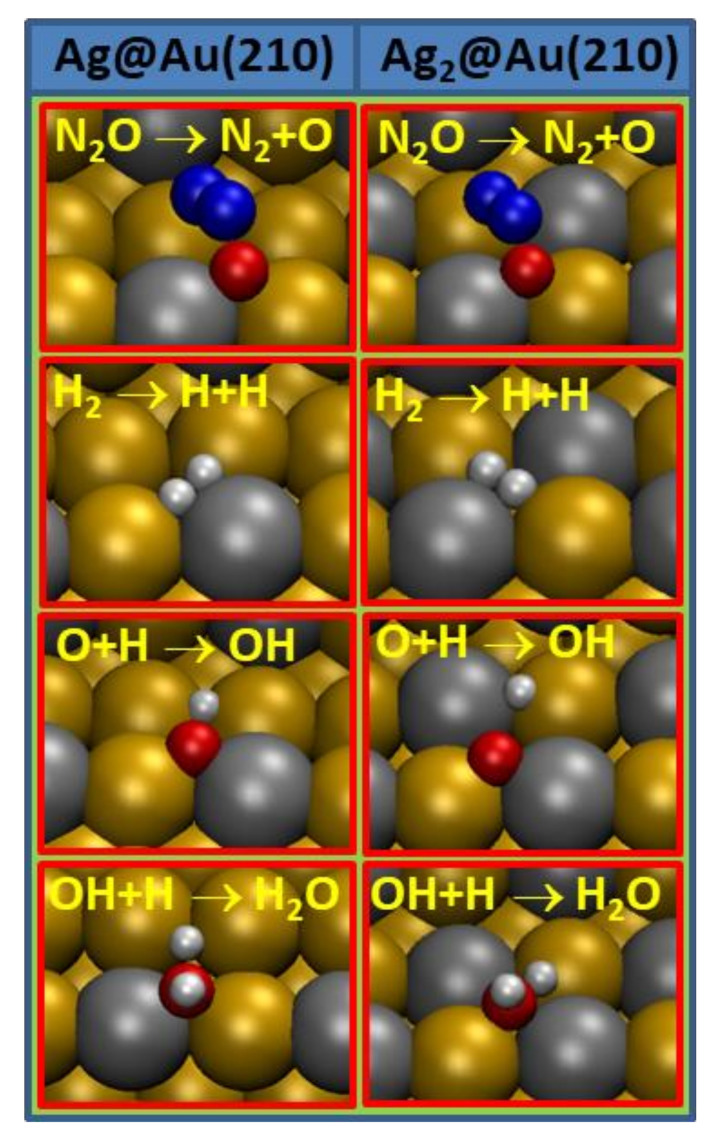
Transition states structures for the steps involved in the N_2_O hydrogenation on Ag@Au(210) and Ag_2_@Au(210) surfaces. Red color is used for oxygen, white for hydrogen, blue for nitrogen, grey for silver and ochre for gold atoms.

From the energies and vibrational frequencies of the IS, TS and FS, the reaction energies and activation energy barriers were obtained by applying Equations (11) and (12), respectively. The values for these quantities, as well as the vibrational frequencies and the estimated values for the rate constant at different temperatures used in the experiments [23], calculated using Equation (13), are given in Table 3. The transition state geometries for each reaction step on Ag@Au(210) and Ag_2_@Au(210) surfaces are depicted in Figure 4.

Let us now check the results obtained for each individual reaction step. As can be seen, the N_2_O dissociation has to surpass activation energy barriers of 0.84 eV on the Ag@Au(210) surface and of 0.69 eV on the Ag_2_@Au(210) surface. Thus, the increase in the silver content in the surface has a positive effect in the catalysis, lowering the activation barriers. Note that the activation energy barrier on the Ag@Au(210) surface is close to that on pure Au(321), i.e., 0.88 eV [45]. Yet, these moderate reaction barriers are considerably higher than the N_2_O adsorption energy in the same surfaces (see Table 1), leading us to predict that the desorption of N_2_O chemical will be an important factor in the nitrous oxide hydrogenation on the surfaces studied here. Moreover, the N_2_O dissociation reaction is thermodynamically very favorable on both surfaces, −0.43 eV on the Ag@Au(210) surface and −0.62 eV on the Ag_2_@Au(210) surface.

If we combine the results obtained here for the N_2_O dissociation with the low adsorption energy of N_2_ on both surfaces and, therefore, the easy desorption of N_2_ from those surfaces (E_act_ of 0.15 eV on Ag@Au(210) and 0.17 eV on Ag_2_@Au(210)), it is expected that the O atoms will accumulate on the surface if they are not eliminated. Furthermore, the reaction rate constant values, estimated from the activation energy barriers and vibrational frequencies, are consistent with the values obtained for the activation energy barriers. Thus, the rate constant presents high values at any temperature examined in this work for N_2_ desorption, while these are moderate for the N_2_O dissociation only at the higher temperatures considered. Interestingly, the rate constant for the N_2_O dissociation on Ag_2_@Au(210) surface displays substantial values for the temperature range considered in the experiments (300 K to 320 K) [23]; see Table 3. In the chemical process studied here, the N_2_O hydrogenation, the O adatoms are removed from surface by their hydrogenation toward water. Firstly, H_2_ has to be dissociated on the surface to provide H adatoms which react with the O adatoms to form hydroxyl on the surface and further water through new hydrogenation. Molecular hydrogen is only physisorbed on both Ag_x_@Au(210) surfaces (see Table 1); therefore, this dissociation has to follow an Eley–Rideal mechanism (dissociative adsorption). The hydrogen dissociation has to surpass moderate reaction barriers of ~0.6 eV on both surfaces and the values for the reaction rate constant estimated here forecast high dissociation velocities at any experimental temperature (300 K to 320 K [23]).

**Table 3:** Activation energies ($E_{act}^{ZPVE}$, eV), frequencies for the vibrational modes (cm^−1^) of the TS, length of the bond breaking or forming in the TS (Å), reaction rate constants at *T* = 200 K, 225 K, 250 K, 275 K, 300 K, 325 K, 350 K and 400 K (k, s^−1^ or mol^−1^ s^−1^), reaction energies ($E_{react}^{ZPVE}$, eV), and imaginary frequencies (cm^−1^) for the steps involved in the N_2_O hydrogenation on Ag@Au(210) and Ag_2_@Au(210) surfaces.

**Ag@Au(210) Surface**						
**Elementary Step**	**Vibrational Modes ^a^**	**Bond Length ^b^**	$E_{act}^{ZPVE}$	*k*	$E_{react}^{ZPVE}$	**Imaginary Frequency**
N_2_O* → N_2_* + O*	1858; 554	1.58	0.84	4.8 × 10^−10^/1.1 × 10^−7^/9.0 × 10^−6^/3.3 × 10^−4^/ 6.8 × 10^−3^/8.8 × 10^−2^/8.0 × 10^−1^/3.0 × 10^1^	−0.43	503
H_2_* → H* + H*	1176; 1000; 725; 557	1.01	0.61	2.4 × 10^−4^/1.1 × 10^−2^/2.1 × 10^−1^/2.4 × 10^0^/ 1.8 × 10^1^/9.8 × 10^1^/4.2 × 10^2^/4.3 × 10^3^	0.13	579
O* + H* → OH*	1183; 1040	1.91	0.32	4.9 × 10^4^/4.3 × 10^5^/2.5 × 10^6^/1.1 × 10^7^/ 3.5 × 10^7^/9.9 × 10^7^/2.4 × 10^8^/1.0 × 10^9^	−1.55	2.53
OH* + H* → H_2_O*	3679; 778; 680; 522	1.57	0.34	8.0 × 10^3^/8.0 × 10^4^/5.1 × 10^5^/2.3 × 10^6^/ 8.3 × 10^6^/2.5 × 10^7^/6.3 × 10^7^/2.9 × 10^8^	−0.88	856
N_2_* → N_2_ + *	—	—	0.15	2.0 × 10^9^/5.1 × 10^9^/1.1 × 10^10^/2.0 × 10^10^/ 3.2 × 10^10^/4.9 × 10^10^/7.0 × 10^10^/1.2 × 10^11^	0.15	—
H_2_O* → H_2_O + *	—	—	0.50	2.5 × 10^−1^/6.5 × 10^0^/8.7 × 10^1^/7.2 × 10^2^/ 4.2 × 10^3^/1.8 × 10^4^/6.4 × 10^4^/4.8 × 10^5^	0.50	—
**Ag_2_@Au(210) Surface**						
N_2_O* → N_2_* + O*	1825; 565	1.56	0.69	3.3 × 10^−7^/2.9 × 10^−5^/1.1 × 10^−3^/2.1 × 10^−2^/ 2.5 × 10^−1^/2.1 × 10^0^/1.3 × 10^1^/2.5 × 10^2^	−0.62	489
H_2_* → H* + H*	1476; 925; 679; 503	1.05	0.57	2.2 × 10^−3^/7.8 × 10^−2^/1.3 × 10^0^/1.3 × 10^1^/ 9.0 × 10^1^/4.5 × 10^2^/1.8 × 10^3^/1.6 × 10^4^	0.07	525
O* + H* → OH*	1674; 524	1.93	0.29	2.4 × 10^5^/1.7 × 10^6^/7.9 × 10^6^/2.9 × 10^7^/ 8.3 × 10^7^/2.1 × 10^8^/4.5 × 10^8^/1.6 × 10^9^	−1.51	314
OH* + H* → H_2_O*	3663; 1330; 764	1.64	0.30	6.1 × 10^4^/4.7 × 10^5^/2.4 × 10^6^/9.4 × 10^6^/ 2.9 × 10^7^/7.5 × 10^7^/1.7 × 10^8^/6.6 × 10^8^	−0.85	257
N_2_* → N_2_ + *	—	—	0.17	1.7 × 10^9^/5.4 × 10^9^/1.3 × 10^10^/2.8 × 10^10^/ 5.3 × 10^10^/9.1 × 10^10^/1.4 × 10^11^/3.0 × 10^11^	0.17	—
H_2_O* → H_2_O + *	—	—	0.47	2.5 × 10^−1^/6.5 × 10^0^/8.7 × 10^1^/7.2 × 10^2^/ 4.2 × 10^3^/1.8 × 10^4^/6.4 × 10^4^/4.8 × 10^5^	0.47	—

^a^ Only the vibrational modes above 500 cm^−1^ are shown. ^b^ Length of the bond breaking or forming in the transition state.

A similar value to those obtained here was also attained for the activation energy barrier of hydrogen dissociation on the pure Au(321) surface, the reaction following also an Eley–Rideal mechanism in that pure surface [45]. Combining both results, one can then suggest that the hydrogen dissociation is almost equally favorable for pure and silver doped gold surfaces. However, the N_2_O dissociation occurs preferably on regions where silver atoms are present in the (210) facets and presents higher adsorption energy than hydrogen. Therefore, hydrogen and nitrous oxide have a preference for different adsorption and dissociation sites as it was suggested from experimental findings, with water being formed in the interface between H* and O* rich regions [23].

After the hydrogen and N_2_O dissociations on the surface, the H^*^ adatoms react with O^*^ adatoms to form hydroxyl, and, through new hydrogenation of the hydroxyl, water is formed. Both hydrogenations present low activation energy barriers (~0.3 eV, see Table 3) on the surfaces studied in this work, which lead to high values for the rate constants at any of the temperatures considered here. Water formed from the O* hydrogenation is further desorbed from the surface; the desorption of this species has to surpass activation energy barriers of about ~0.5 eV on the Ag@Au(210) and Ag_2_@Au(210) surfaces. The values obtained for the water desorption rate constant from both surfaces follow those of the activation barriers, i.e., they are high for most of the temperatures considered in this work.

## 4. Discussion

Having described the most favorable paths for the steps shown in Equations (1)–(8), along with the adsorption of reactants and products, we are now in a position to elucidate the full reaction mechanism for N_2_O hydrogenation on silver doped Au(210) surfaces.

In the first step, the adsorption of N_2_O species on the silver atoms of the doped Au(210) surfaces is not significantly influenced by the silver content of the surface. Note that nitrous oxide does not adsorb in pure gold surfaces as Au(321), which corresponds to a very reactive surface within those formed exclusively by gold [45]. Yet, the N_2_O dissociation is considerably more favorable on the Ag_2_@Au(210) model surface than on Ag@Au(210), and the activation energy in the last one is closer to that on pure Au(321) surface [45]. Therefore, the silver content on the surface is a crucial aspect in the N_2_O dissociation catalysis, and the decomposition of this species has to occur on the Au(210) facets doped with silver atoms. On the other hand, hydrogen dissociation has almost the same probability of occurring on silver doped gold surfaces as on pure gold surfaces, such as Au(321), where this species is only physisorbed. Thus, hydrogen dissociation has to occur through the dissociative adsorption on the surface, being not accessible for hydrogen the regions with silver atoms due to the stronger N_2_O adsorption. Combining the results for the hydrogen and nitrous oxide adsorption and dissociation, this allows us to conclude that the dissociation of these species occurs in different regions of the catalyst, as it was suggested from the experimental results [23]. Note that O* is more strongly adsorbed on the surface and its spillover is not probable, and, therefore, H* has to spill over to the N_2_O dissociation sites edge to form water.

After the N_2_O and H_2_ dissociations, N_2_ formed by nitrous oxide dissociation desorbs easily from the surface, while O* and H* atoms react to form hydroxyl, which, through new hydrogenation, forms water. O* and H* atoms have to react in the edge between the regions where N_2_O and H_2_ are dissociated, which is in agreement with the experimental observation of an interface with H* and O* adatoms in the edges of (210) facets [23]. Hydroxyl and water formation present low activation energy barriers. That is, water is easily formed, which desorbs from the silver doped gold surfaces, having to surpass moderate-low barriers.

**Figure 5:**
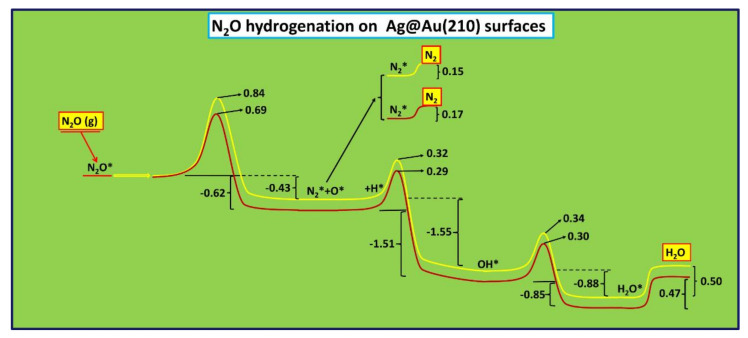
Comparison of the N_2_O hydrogenation reaction profiles on Ag@Au(210) (highlighted in yellow) and Ag_2_@Au(210) (highlighted in red) surfaces. Activation and reaction energies are given in eV.

Comparing the activation energy barriers for all the reaction steps, it can be seen that the N_2_O dissociation is the rate determining step of the global catalytic process, and this step occurs preferably on silver doped gold surfaces with (210) miller indices. A comparison between the reaction profiles on Ag_2_@Au(210) and Ag@Au(210) surfaces of all the steps involved in N_2_O hydrogenation can be seen in Figure 5.

The dispersion of the silver dopant atoms is also a crucial aspect in the catalysis because O* atoms are strongly adsorbed in the surface; this blocks their possible migration toward regions where hydrogen is dissociated, and therefore, it impedes their hydrogenation. Thus, if large silver patches are present in the surface, the O accumulation is foreseen, while if silver atoms are well dispersed, O adatoms can be easily hydrogenated.

The results obtained in this work, that is, the reaction mechanism and the preferable reaction sites for each reactant evolution, are important advances in the understanding of the N_2_O hydrogenation on silver doped gold surfaces which can be further used to design more efficient catalysts for the N_2_O reduction to atmospheric N_2_. As mentioned in the Introduction, the elimination of this species in anthropogenic sources is a crucial aspect in the reduction of greenhouse gases emissions due to the high contribution of this gas to the greenhouse effect [1,2]. It is especially noticeable the fact that N_2_O and H_2_ dissociate on different surface sites, which implies that O* and H* have to react in the edge between both dissociation sites to form water, and if silver sites are large patches, surface poison by O* adsorption is predictable. Therefore, silver atoms have to be well dispersed in the catalytic surface to avoid O* accumulation as was previously suggested by experimental results [23].

## 5. Conclusions

Periodic density functional theory calculations were carried out with the aim of providing insights into the mechanism of N_2_O hydrogenation on silver doped Au(210) surfaces. This computational approach allowed us to establish the reaction mechanism to obtain the contribution of each reaction site to the catalysis as well as the determination of the most stable catalytic surfaces through its geometrical optimization. The aspects mentioned before cannot be easily stated only through experimental measurements. Hence, the most favorable reaction paths for the steps involved in N_2_O hydrogenation were determined on two silver doped gold model surfaces with different silver contents. Activation energy barriers, reaction energies and rate constants were obtained from the energetic quantities for the most favorable path of each reaction step of those involved in N_2_O hydrogenation. Putting together all the reaction steps and considering their energetic quantities, it was possible to elucidate the most favorable reaction mechanism for the N_2_O hydrogenation on silver doped gold surfaces, which consists of the N_2_O and H_2_ dissociations in different regions of the catalytic surface and the further reaction of O and H adatoms into the formation of water. N_2_ and H_2_O species easily desorb from the surface making the N_2_O dissociation step the one determining the global reaction rate. Moreover, a good dispersion of the silver dopant atoms in the gold matrix is crucial, due to the strong adsorption of oxygen atoms in N_2_O dissociation sites, which prevents its migration toward sites where its hydrogenation occurs. Thus, the geometrical optimization of Ag@Au(210) surfaces with different silver contents leads to the obtention of a most stable bimetallic surface with silver atoms on the terrace and a step surrounded by gold atoms. This surface comprises the active sites for the N_2_O dissociation, while H_2_ dissociates on pure gold or bimetallic surfaces with a lower silver content.
